# Reproducibility of ACT, A simple-self-administered sCreening Tool for diabetic peripheral neuropathy

**DOI:** 10.3389/fneur.2026.1847401

**Published:** 2026-07-28

**Authors:** Hoda Gad, Einas Elgassim, Srividya Bennabhaktula, Areej Baraka, Khaled Baagar, Andrej Orszag, Leif Erik Lovblom, Bruce A. Perkins, Rayaz A. Malik

**Affiliations:** 1 Lunenfeld-Tanenbaum Research Institute, Mount Sinai Hospital, Toronto, ON, Canada; 2 Research Department, Weill Cornell Medicine-Qatar, Doha, Qatar; 3 Endocrinology Department, Hamad Medical Corporation, Doha, Qatar; 4 Institute of Health Policy, Management and Evaluation, University of Toronto, Toronto, ON, Canada; 5 Biostatistics Department, University Health Network, Toronto, ON, Canada; 6 Department of Medicine, Division of Endocrinology, University of Toronto, Toronto, ON, Canada; 7 Institute of Cardiovascular Medicine, University of Manchester, Manchester, United Kingdom

**Keywords:** ACT, DPN, MNSI, reproducibility, screening

## Abstract

**Introduction:**

Diabetic Peripheral Neuropathy (DPN) is associated with pain and increased risk of foot ulceration and lower-limb amputation. Most people remain undiagnosed due to the lack of a simple, rapid, and reliable screening tool suitable for primary care. We evaluated the reproducibility of a short, self-administered screening tool (ACT) for DPN compared with the Michigan Neuropathy Screening Instrument Questionnaire (MNSI_q_) and examination (MNSI_e_).

**Methods:**

In this observational study, 126 adults with type 1 or type 2 diabetes were recruited from an outpatient endocrinology clinic. Participants completed the ACT_questionnaire (q) + examination (e)_ which can be summed to create a total_q + e_ score of 0–20, and MNSI_q + e_ at baseline and after 14 days. Reproducibility was assessed using intraclass correlation coefficients (ICC) for continuous scores and Cohen’s kappa (*κ*) for categorical reproducibility.

**Results:**

ACT_q + e_ demonstrated good reproducibility with an ICC = 0.85 (ICC = 0.78 for ACT_q_; ICC = 0.88 for ACT_e_) which was comparable to ICC = 0.82 for MNSI_q_; ICC = 0.75 for MNSI_e_; ICC = 0.85 for MNSI_q + e_. Dichotomized classification of the overall ACT showed strong repeatability with κ = 0.83 (*κ* = 0.61 ACT_q_ and moderate-to-strong repeatability for ACT_e_ with *κ* = 0.78). Symptoms showed variable agreement, with the strongest reproducibility for burning and pins-and-needles sensations. The sensory examination components of ACT_e_ demonstrated strong agreement (*κ* = 0.81).

**Discussion:**

The overall ACT tool demonstrated good 14-day reproducibility compared to the overall MNSI_q + e_. The brevity, self-administered format, and minimal reliance on clinician expertise, support the utility of ACT as a practical screening instrument for DPN in busy, resource-limited clinical settings.

## 1 Introduction

Diabetic peripheral neuropathy (DPN) contributes to painful neuropathy with reduced quality of life, and an increased risk of foot ulceration and lower-limb amputation (1). Globally, DPN affects up to 50% of individuals with diabetes, presenting a massive healthcare burden that scales with the rising worldwide prevalence of diabetes (2). Unfortunately, most patients with DPN go un-diagnosed due to non-specific symptoms that go unreported in routine clinical encounters (3). Screening and early detection are essential to reduce morbidity and improve outcomes (4).

There is currently no test for DPN detection that is simple, rapid, and feasible in busy clinical settings. Neurological examination, sensory testing and electrophysiological studies require specialized equipment or training, are time-intensive, and are impractical in primary care (3). Furthermore, many commonly used clinical scales and questionnaires for DPN are subjective and heavily dependent on clinician interpretation, raising concerns about their validity when applied outside research settings. Such instruments have been developed and validated in large population studies where resources and trained personnel are available, but assessment of their performance in routine clinical practice has been limited (3).

A number of questionnaires and scoring instruments such as the Michigan Neuropathy Screening Instrument (MNSI), Toronto Clinical Neuropathy Score (TCNS), and Neuropathy Impairment Score are available, but can be lengthy, complex to score, or reliant on examiner expertise (3). Furthermore, diagnostic accuracy varies widely across tools, and systematic reviews highlight the ongoing need for practical, efficient, and reliable screening methods that can be easily implemented at the point of care (5).

To address these limitations, we developed a short, self-administered screening tool (ACT) to facilitate DPN detection in primary care and resource-limited settings (6, 7). This tool was designed to be simple for patients to complete, easy to score, and less reliant on clinician interpretation compared with traditional instruments (6, 7).

In the present study, ACT was administered to individuals with diabetes at baseline and after two weeks compared to the reference-standard MNSI to evaluate the 14-Day reproducibility of ACT.

## 2 Methods

### 2.1 Study design and setting

Adults with diabetes attending the outpatient endocrinology clinic at Hamad Medical Corporation (HMC) were recruited. Ethical approval was obtained from the Institutional Review Boards of Weill Cornell Medicine-Qatar 24–00029 and Hamad Medical Corporation MRC-01-24-543. The study was conducted in accordance with the principles of the Declaration of Helsinki. Written informed consent was obtained from all participants prior to enrollment. This study was registered at ClinicalTrials.gov (Identifier: NCT07163000).

### 2.2 Participants

A total of 150 participants with a confirmed diagnosis of type 1 diabetes mellitus (T1DM) or type 2 diabetes mellitus (T2DM) were consecutively recruited. Eligible participants were adults aged 18 years or older who attended the outpatient endocrinology clinic and completed the study questionnaire. To evaluate the test–retest reliability of the screening instrument, participants were asked to return after 14 days—a duration selected to minimize recall bias while ensuring clinical stability (8) to repeat both MNSI and ACT.

### 2.3 The ACT tool

The ACT tool consists of two components: a patient-administered questionnaire (ACT_q_) and a clinician based sensory examination (ACT_e_) with a predefined cut-off score identifying abnormality of >4 for ACT_q_, and ≥2 for ACT_e_ (7).

#### 2.3.1 Patient-administered questionnaire

1) Symptom description: Six items assess the presence of sensations in the hands and/or feet, including burning, tingling, electric shocks, numbness, pins and needles, or other unusual sensations.
2) Symptom severity: One item rates how bothersome symptom have been over the past two weeks on a 1–10 scale, where 1 indicates “not at all” and 10 “most bothersome”.
3) Symptom symmetry: One item identifies the location of sensations on a body diagram and whether they are symmetrical.
4) Nocturnal exacerbation: One item determines if sensations worsen at night.
5) Risk factors: Six items capture potential risk factors for diabetic peripheral neuropathy (DPN), including diabetes or prediabetes, long-term medication use, obesity, restricted diets (e.g., vegan, vegetarian, gluten-free), smoking, and alcohol consumption. This section informs risk assessment and follow-up planning.

#### 2.3.2 Sensory examination

Administered only if symptoms are reported (ACT_q_ > 0) and includes a pin-prick test for hypoesthesia and a cotton wool/brush test for allodynia in both feet.

The total possible score for the ACT_q_ ranges from 0 to 14, while the ACT_e_ ranges from 2 to 6 with the overall ACT_q + e_ score ranging from 0 to 20.

MNSI was used as the validated reference-standard to screen for DPN and compared to ACT with a predefined cut-off scores of ≥4 for MNSI_q_ and ≥2.5 for MNSI_e_ (9). MNSI was selected as the reference standard as it is a validated, sensitive and specific and widely accepted tool for identifying DPN in large epidemiological and smaller cohort studies. The score for the MNSI_q_ ranges from 0 to 13, while the MNSI_e_ ranges from 0 to 10 and the MNSI_q + e_ score ranges from 0 to 23.

### 2.4 Sample size determination

Using the 30:5 item-to-respondent ratio approach (10), a minimum of 150 participants was required to validate the 5-item ACT questionnaire.

Sample size was estimated based on reliability and agreement parameters. Assuming an expected intraclass correlation coefficient (ICC) of 0.80, a two-sided *α* level of 0.05, and statistical power between 80%, the required sample size was estimated to range between 40 and 50 participants. For agreement assessment using Cohen’s kappa, assuming an expected kappa value of 0.70, an α level of 0.05, and 80% power, the estimated required sample size ranged between 80 and 120 participants. To ensure adequate power for both reliability measures, all participants with complete follow-up data were included in the reproducibility analysis. Of the 150 enrolled participants, 126 completed both baseline and 14-day assessments and were therefore included in the final reproducibility analysis, exceeding the minimum requirements for ICC-based calculations and meeting the required range for kappa-based analysis.

### 2.5 Data collection and clinical assessments

At baseline, demographic and clinical data were collected, and clinical and laboratory parameters were obtained from medical records including total cholesterol, triglycerides, high-density lipoprotein cholesterol, low-density lipoprotein cholesterol, glycated hemoglobin A1c (HbA1c) and estimated glomerular filtration rate (eGFR).

Participants attended a baseline visit and after 14 days (*n* = 126) to undergo re-administration of the ACT and MNSI questionnaires to assess test–retest reproducibility.

### 2.6 Statistical analysis

All statistical analyses were performed using IBM SPSS Statistics software Version 30.0. Normality of the data was assessed using the Shapiro–Wilk test and by visual inspection of the histogram and a normal Q-Q plot. Data are expressed as mean ± SD for the normally distributed variables and as median [IQR] or median (Range) for the skewed variables, or *n* (%) for categorical variables.

Reproducibility was evaluated using the intraclass correlation coefficient (ICC) for the continuous variables, and Cohen’s kappa with 95% CI for categorical or dichotomized variables [both with corresponding 95% confidence intervals (CIs)].

ICC was applied to total scores of continuous data which included: MNSI_q + e_, MNSI_q_, MNSI_e_, ACT_q + e_, ACT_q_, ACT_q_ Q1 on symptoms, severity of symptoms Q1 of ACT_q_, DPN risk factors Q5 of ACT_q_, ACT_e._ Cohen’s kappa was applied to dichotomized data as the MNSI_q + e_, MSNI_q_, MNSI_e_ and ACT_q + e_, ACT_q_, and ACT_e_ as binary data to define patients with and without DPN based on the predefined cut-off. Kappa statistics were also computed for the individual components of the MNSI_q_ (Q1–3, Q5–9, Q11–15) and MNSI_e_. Furthermore, Kappa was applied to individual items of Q1 of ACT_q_ (burning, tingling, electric shock, numbness, pins and needles, other unusual sensation), Q3 ACT_q_ on distribution/symmetry, Q4 of ACT_q_ on nocturnal symptoms. ICC values were interpreted according to established thresholds: <0.50 poor reliability, 0.50–0.75 moderate reliability, 0.75–0.90 good reliability, and >0.90 excellent reliability, with interpretation based on both the point estimate and its 95% CI (11). Cohen’s kappa values were interpreted as follows: 0–0.20 poor, 0.21–0.39 minimal, 0.40–0.59 weak, 0.60–0.79 moderate, 0.80–0.90 strong, and >0.90 almost perfect agreement (12).

### 2.7 Sensitivity analysis

Given the high variability in the kappa coefficient observed for item Q5 of the ACT questionnaire, a sensitivity analysis was conducted to evaluate its impact on internal consistency. The overall Cronbach’s alpha for the questionnaire was (*α* = 0.63) with Q5 included and (α = 0.75) after removal of Q5.

## 3 Results

### 3.1 Participant characteristics

A total of 126 participants aged 52.5 ± 12.4 years, male (62.7%), T2DM (84.9%), T1DM 15.1% with a median duration of diabetes was 14.0 years [5.0, 20.0] and median HbA_1c_ of 7.80 [6.70, 9.30] % were recruited. Most participants had a college level of education (65.9%). Around 86% of participants were classified as stage 2 chronic kidney disease (eGFR 60–89 mL/min/1.73 m^2^), and 2 cases had end-stage renal disease (ESRD) treated with hemodialysis. The mean total cholesterol was 4.34 ± 0.86 mmol/L, mean LDL cholesterol was 2.32 ± 0.81 mmol/L, median HDL cholesterol was 1.20 [1.00, 1.50] mmol/L, and median triglycerides were 1.40 [0.95, 2.10] mmol/L. Median vitamin B12 level was 364 [265, 489] pmol/L. Participants were defined as having DPN based on a median MNSI_q_ score 3.0 (Min–Max 0–9), median MNSI_e_ score 2.5 (Min-Max 0–8), median ACT_q_ score 5.0 (Min–Max 2–11), and median ACT_e_ score 2.0 (Min–Max 0–8) (Table 1).

**Table 1:** Baseline characteristics of patients.

Variables (*n* = 126)	
Age (years)	52.5 ± 12.4
Gender, *n* (%)	
Male	79.0 (62.7)
Female	47.0 (37.3)
Level of education, *n* (%)	
Primary	10.0 (7.90)
Secondary	16.0 (12.7)
College	83.0 (65.9)
Higher education	9.00 (7.10)
Duration of diabetes (years)	14.0 [5.00, 20.0]
Type of diabetes, *n* (%)	
T1DM	19.0 (15.1)
T2DM	107 (84.9)
Weight (kg)	97.2 [69.0, 89.0]
A1c (%)	7.80 [6.70, 9.30]
Kidney disease by eGFR (ml/min/1.73m^2^), *n* (%)	
Stage 1: eGFR ≥ 90	8.00 (6.40)
Stage 2: eGFR 60–80	108 (86.4)
Stage 3: eGFR 30–59	6.00 (4.80)
Stage 4: eGFR 15–29	1.00 (0.80)
Stage 5: eGFR <15	2.00 (1.60) ^+^
Total Cholesterol (mmol/L)	4.34 ± 0.86
LDL (mmol/L)	2.32 ± 0.81
HDL (mmol/L)	1.20 [1.00, 1.50]
TG (mmol/L)	1.40 [0.95, 2.10]
Vit B12 (pmol/L)	364 [265, 489]
Questionnaires	
MNSI_q_	3.00 (9.00) 0.00–9.00
MNSI_e_	2.50 (8.00) 0.00–8.00
ACT_q_	5.00 (9.00) 2.00–11.0
ACT_e_	2.00 (6.00) 0.00–8.00

T1DM: type 1 diabetes mellitus, T2DM: type 2 diabetes mellitus, A1c: glycated hemoglobin, eGFR: estimated glomerular filtration rate, LDL: low-density lipoprotein, HDL: high-density lipoprotein, TG: triglycerides, Vit B12: vitamin B12, MNSI: Michigan neuropathy screening instrument, q: questionnaire, e: examination, ACT: A simple sCreening Tool. Cut off for abnormal is as follows: MNSI_q_ >4, MNSI_e_ ≥2.5, ACT_q_ >4 and ACT_e_ >3. Data is summarized as mean ± SD for the parametric variables and as median [Q1, Q3] or the non-parametric variables. Questionnaire data is summarized as median (range) and Min–Max. Frequency of the missing data for the following variables: Weight (*n* = 2), level of education (*n* = 8), duration of diabetes (*n* = 11), A1c (*n* = 1), eGFR (*n* = 1), TC (*n* = 1), LDL (*n* = 1), HDL (*n* = 1), TG (*n* = 2), B12 (*n* = 9), ACT_e_ (*n* = 1*). * ACT_e_ (foot examination section) was not performed in one patient due to a dressing for severe neuropathy–related burn. + Two patients were on hemodialysis.

### 3.2 Reproducibility of continuous scores

Intraclass correlation coefficients (ICC) demonstrated good reproducibility for the ACT_q + e_ score (ICC = 0.85). ACT_q_ showed good reliability (ICC = 0.78), with its subcomponents indicating good reproducibility for Q1 (total symptom score; ICC = 0.82), moderate reproducibility for Q2 (symptom severity; ICC = 0.67), and good reproducibility for Q5 (DPN risk factors; ICC = 0.76). ACT_e_ demonstrated the highest reproducibility among the ACT components (ICC = 0.88). MNSI_q + e_ showed good reproducibility (ICC = 0.85), with MNSI_q_ (ICC = 0.82) and MNSI_e_ (ICC = 0.75) both also demonstrating good reproducibility (Table 2).

**Table 2:** Reproducibility of continuous scores by intraclass correlation coefficient (ICC).

Scale	Mean ± SD (baseline)	Mean ± SD (14 Days)	ICC (95% CI)
**Overall ACT**_**q + e**_ **score**	8.02 ± 2.89	7.60 ± 2.99	0.85 (0.79–0.89)
**ACT**_**q**_	5.60 ± 2.35	5.21 ± 2.42	0.78 (0.69–0.84)
ACT Q1 on symptoms^*^	1.89 ± 1.64	1.67 ± 1.61	0.82 (0.750–0.87)
Q2. Severity	3.82 ± 3.11	3.46 ± 3.06	0.67 (0.56–0.75)
ACT Q5 on risk factors for DPN^*^	2.72 ± 0.72	2.66 ± 0.77	0.76 (0.67–0.82)
**ACT**_**e**_	2.42 ± 1.13	2.38 ± 1.05	0.88 (0.85–0.92)
**Overall MNSI**_**q + e**_	5.66 ± 3.36	5.59 ± 3.41	0.85 (0.79–0.89)
MNSI_q_	2.89 ± 2.41	2.75 ± 2.30	0.82 (0.75–0.87)
MNSI_e_	2.77 ± 1.56	2.73 ± 1.62	0.75 (0.66–0.82)

q: questionnaire, e: examination, ACT: A simple sCreening Tool, MNSI: Michigan neuropathy screening instrument. *Details of the Dichotomized responses are summarized in Table 3. MNSI_q+e_ analysis included *n* = 126 valid cases. ACT_q_ analysis included *n* = 126 valid cases. ACT_e_ analysis included *n* = 125 valid cases, foot examination section was not performed in one patient due to a dressing for severe neuropathy–related burn. Values in bold represent total questionnaire scores; non-bolded values represent individual item scores.

### 3.3 Reproducibility of dichotomized scores

When dichotomized scores were analyzed between baseline and 14 days, Overall ACT_q + e_ demonstrated strong reproducibility (*κ* = 0.83, 95% CI: 0.67–0.99), while ACT_q_ showed moderate reproducibility (*κ* = 0.61, 95% CI: 0.45–0.76) (see Table 3).

Within ACT_q_ Q1 symptoms, reproducibility ranged from minimal to strong: burning (*κ* = 0.75) and pins and needles (κ = 0.70) showed strong reproducibility; tingling (*κ* = 0.58) moderate reproducibility; electric shock–like pain (*κ* = 0.48) and numbness (*κ* = 0.42) weak to moderate reproducibility; and other unusual sensations (*κ* = 0.34) minimal reproducibility. Symmetry/distribution (Q3) demonstrated moderate reproducibility (*κ* = 0.59), as did nocturnal symptoms (Q4) (*κ* = 0.61).

**Table 3:** Reproducibility of dichotomized scores by Cohen’s weighted kappa coefficient.

Scale (baseline vs. 14 days)	κ (95% CI)
The overall reproducibility for the dichotomized classification of DPN^+^ vs. DPN^−^	
Overall ACT_q + e_	0.83 (0.67–0.99)
Overall MNSI_q + e_	0.64 (0.46–0.83)
Reproducibility for the dichotomized classification of the individual components of ACT	
ACT_q_	0.61 (0.45–0.76)
ACT_q_, Q1. Symptoms	
Q1. Burning	0.75 (0.64–0.88)
Q1. Tingling	0.58 (0.44–0.73)
Q1. Electric shock like pain	0.48 (0.26–0.69)
Q1. Numbness	0.42 (0.26–0.59)
Q1. Pins and needles	0.70 (0.56–0.83)
Q1. Other unusual sensation	0.34 (0.17–0.52)
Q3. Symmetry/distribution	0.59 (0.45–0.73)
Q4. Nocturnal symptoms	0.61 (0.47–0.75)
ACT_q,_ Q5. Risk factors for DPN	
Diabetes^+^	100% agreement
Chronic medication use	0.27 (−0.18–0.71)
Obesity	0.67 (0.54–0.80)
Alternative diet	0.74 (0.40–1.08)
Smoking	0.96 (0.87–1.04)
Alcohol use	0.84 (0.67–1.02)
ACT_e_	0.78 (0.620–0.94)
Pinprick	0.81 (0.68–0.94)
Cotton wool/brush – light touch	0.81 (0.69–0.93)
Reproducibility for the dichotomized classification of the individual components of MNSI	
MNSI_q_^*^	0.66 (0.50–0.82)
Q1. Numbness	0.74 (0.62–0.86)
Q2. Burning pain	0.70 (0.57–0.83)
Q3. Hypersensitivity	0.39 (0.21–0.57)
Q5. Tingling	0.58 (0.40–0.76)
Q6. Allodynia (bed-sheet pain)	0.48 (0.25–0.70)
Q7. Temperature sensitivity	0.47 (0.15–0.78)
Q8. Open sores	0.74 (0.52–0.96)
Q9. Amputation	0.70 (0.48–0.93)
Q11. Leg pain (walking)	0.45 (0.29–0.61)
Q12. Foot sensation	0.43 (0.25–0.60)
Q13. Diagnosis of neuropathy	0.40 (0.10–0.70)
Q14. Dry skin/cracks	0.40 (0.10–0.70)
Q15. History of amputation	0.29 (0.12–0.46)
MNSIe	0.46 (0.30–0.61)
Q1. Foot appearance (R)	0.69 (0.49–0.88)
Q1. Foot appearance (L)	0.69 (0.49–0.88)
Q2. Ulceration (R)	1.00 (1.00–1.00)
Q2. Ulceration (L)	0.66 (0.04–1.28)
Q3. Ankle reflexes (R)	0.33 (0.10–0.57)
Q3. Ankle reflexes (L)	0.31 (0.06–0.55)
Q4. Vibration (R)	0.50 (0.36–0.64)
Q4. Vibration (L)	0.47 (0.33–0.60)
Q5. Monofilament (R)	0.72 (0.56–0.87)
Q5. Monofilament (L)	0.65 (0.48–0.82)

q: questionnaire, e: examination, ACT: A simple sCreening Tool, MNSI: Michigan neuropathy screening instrument. +Kappa could not be computed for diabetes due to lack of variability, where participants indicated the same response. *MNSIe Q4 and 10 are not included in the total score, hence not presented in Table 3. (R): refers to the right foot, (L): refers to the left foot.

For ACT_q_ Q5 risk factors, diabetes showed perfect reproducibility (*κ* = 1.00), chronic medication use minimal reproducibility (*κ* = 0.27), obesity moderate reproducibility (*κ* = 0.67), alternative diet strong reproducibility (κ = 0.74), smoking almost perfect reproducibility (*κ* = 0.96), and alcohol use strong reproducibility (κ = 0.84). ACT_e_ demonstrated moderate to strong reproducibility (*κ* = 0.78, 95% CI: 0.62–0.94), with strong reproducibility for both pinprick (*κ* = 0.81) and light touch using cotton wool/brush (*κ* = 0.81).

Overall MNSI_q + e_ demonstrated moderate reproducibility (*κ* = 0.64, 95% CI: 0.46–0.83). MNSI_q_ showed moderate reproducibility (*κ* = 0.66, 95% CI: 0.50–0.82), with item-level reproducibility ranging from minimal to strong: numbness (*κ* = 0.74), burning pain (κ = 0.70), hypersensitivity (κ = 0.39), tingling (*κ* = 0.58), allodynia (*κ* = 0.48), temperature sensitivity (*κ* = 0.47), open sores (*κ* = 0.74), amputation (*κ* = 0.70), leg pain on walking (*κ* = 0.45), foot sensation (*κ* = 0.43), neuropathy diagnosis (*κ* = 0.40), dry skin/cracks (κ = 0.40), and history of amputation (κ = 0.29).

MNSI_e_ demonstrated weak reproducibility (κ = 0.46, 95% CI: 0.30–0.61). Item-level reproducibility ranged from poor to very strong: foot appearance (right and left, κ = 0.69), ulceration (right κ = 1.00; left κ = 0.66), ankle reflexes (right κ = 0.33; left κ = 0.31), vibration (right κ = 0.50; left κ = 0.47), and monofilament (right κ = 0.72; left κ = 0.65).

## 4 Discussion

The ACT tool demonstrated excellent reproducibility across multiple domains. The overall ACT (ACT_q_ and ACT_e_) provided consistent scores with an ICC of 0.85; while using the ACT diagnostic algorithm the presence or absence of neuropathy provided consistent classification with a kappa of 0.83. In addition, the self-administered ACT_q_ component showed strong test–retest reliability, and the clinician-administered ACT_e_ component exhibited high inter-rater reliability. Notably, this reproducibility was comparable to that observed for the classification of neuropathy using the MNSI scoring system, including its individual MNSI_q_ but not MNSI_e_ components.

Reliability is a fundamental psychometric requirement for screening tools intended for longitudinal monitoring or repeated clinical application. An ICC above 0.75 indicates good reliability and supports clinical usability (11). In our study, ACT_q_ and ACT_e_ both met or exceeded this threshold, reinforcing the internal stability of the instrument. Importantly, reproducibility was assessed using both continuous and dichotomized scoring approaches, reflecting real-world clinical application where patients are classified with or without DPN.

The MNSI is among the most widely used clinical instruments for DPN screening and has demonstrated acceptable diagnostic performance in populations with type 1 and type 2 diabetes (9, 13). Our findings showed comparable reproducibility between ACT_q_ versus MNSI_q_, and ACT_q + e_ versus MNSI_q + e_, supporting the construct validity of ACT as a screening tool. On the other hand, ACT_e_ showed moderate to strong repeatability compared to a weak repeatability of MNSI_e_. Indeed, while MNSI has been extensively evaluated for diagnostic accuracy, formal short-interval test–retest reproducibility has not been evaluated in many validation studies. Similarly, other established instruments such as the Toronto Clinical Neuropathy Score (TCNS) and the Neuropathy Impairment Score were developed to assess severity and diagnostic correlation rather than short-term repeatability (14, 15). In this context, our study explicitly evaluates the temporal stability of ACT compared to the reference-standard of MNSI, addressing an important but often underreported psychometric property.

At the item level, reproducibility varied across neuropathic symptom descriptors. Burning and pins-and-needles sensations demonstrated moderate agreement, whereas electric shock–like sensations and numbness showed weaker agreement. Neuropathic symptoms in DPN are known to fluctuate in intensity and character over time, which may partially explain this variability (16). Burning, tingling, and electric shock–like pain are well-recognized features of painful DPN and are incorporated into consensus definitions and diagnostic frameworks (16, 17). Therefore, moderate repeatability for subjective symptoms likely reflects individual biological variability rather than measurement instability (18). Similarly, MNSI individual components showed variable reproducibility with numbness and burning demonstrating the highest reproducibility.

The examination components of ACT_e_ (pinprick and light-touch testing) demonstrated strong agreement. Current recommendations from the ADA advocate annual screening for DPN using simple bedside sensory assessments to identify patients at risk for ulceration and amputation (1). However, implementation in busy or resource-limited settings remains inconsistent. Indeed, as highlighted by an international consensus on primary care screening, standard sensory assessments like the 10 g monofilament are low-cost and accessible but exhibit limited clinical accuracy in early DPN, often only capturing patients with advanced neuropathy at high risk of foot ulceration (6). Conversely, while comprehensive diagnostic questionnaires offer alternative validation, they are frequently restricted by complex scoring structures and lengthy administration times that are logistically impractical during rapid clinical encounters (6). By combining a self-administered symptom section with a simplified sensory examination, we believe ACT will facilitate adherence to guideline-based screening while minimizing clinician burden. Of note, removal of Q5 improved internal consistency (Cronbach’s *α* increasing from 0.63 to 0.75), suggesting that this item may reduce overall scale coherence. Although the ACT_q_ demonstrated acceptable reliability overall, this finding indicates that further refinement and formal validation of a modified version of the questionnaire without Q5 may be warranted.

Systematic evaluations of DPN screening tools have highlighted substantial heterogeneity in methodology, diagnostic thresholds, and psychometric reporting, with limited emphasis on reproducibility across short intervals (8). The practical feasibility of a tool relies heavily on bridging the clinician-time deficit. By utilizing a brief tool that actively involves the patient in self-screening, ACT minimizes the active time commitment required by a physician to arrive at a clinical baseline, aligning with recommended frameworks to mitigate the widespread (~80%) under-diagnosis of peripheral neuropathy globally (6). Our study addresses this gap by demonstrating that ACT maintains good reliability over time, which is crucial for follow-up, monitoring progression, or evaluating response to intervention.

Several limitations merit consideration. MNSI was used as a comparator to the ACT tool to evaluate the latter’s reproducibility and whilst widely used, it is not equivalent to objective measures of neuropathy such as nerve conduction studies. Additionally, the study was conducted in a tertiary care setting, which may limit generalizability to primary care populations with milder DPN. Symptom fluctuation over the 14-day interval may also have influenced agreement for subjective items, although this reflects real-world clinical variability.

In summary, ACT demonstrated good reproducibility and acceptable agreement compared with MNSI, with strong performance of its examination components and stable overall scoring. Unlike many existing DPN instruments that primarily report diagnostic validity or severity correlation, our study explicitly established short-term repeatability, strengthening the psychometric foundation of ACT. By demonstrating robust test–retest reliability over a 14-day window, this study addresses a critical gap in the DPN literature, showing that the tool provides stable, dependable metrics that are not confounded by immediate recall bias or daily clinical fluctuations. Given its brevity, self-administered structure, and minimal reliance on examiner expertise, ACT represents a promising, pragmatic screening tool for DPN in busy and resource-limited clinical settings. Crucially, its widespread implementation could streamline outpatient workflows, drastically reduce the clinical burden on primary and secondary care physicians, and facilitate early, proactive intervention for at-risk patients before irreversible complications arise. Further validation against other established screening instruments and more objective measures of DPN is warranted.

## Data Availability

The raw data supporting the conclusions of this article will be made available by the authors, without undue reservation.
